# Epigenetic regulation of polyomavirus JC

**DOI:** 10.1186/1743-422X-10-264

**Published:** 2013-08-23

**Authors:** Hassen S Wollebo, Baheru Woldemichaele, Kamel Khalili, Mahmut Safak, Martyn K White

**Affiliations:** 1 Department of Neuroscience, Center for Neurovirology, Temple University School of Medicine, 3500 N. Broad Street, Philadelphia, PA 19140, USA

**Keywords:** Epigenetic, Acetylation, Transcriptional regulation

## Abstract

**Background:**

Polyomavirus JC (JCV) causes the CNS demyelinating disease progressive multifocal leukoencephalopathy (PML), which occurs almost exclusively in people with immune deficiencies, such as HIV-1/AIDS patients. JCV infection is very common and usually occurs early in life. After primary infection, virus is controlled by the immune system but, rarely when immune function is impaired, it can re-emerge and multiply in the astrocytes and oligodendrocytes in the brain and cause PML. Thus a central question in PML pathogenesis is the nature of the molecular mechanisms maintaining JCV in a latent state and then allowing reactivation.

**Methods:**

Since transcription can be regulated by epigenetic mechanisms including DNA methylation and histone acetylation, we investigated their role in JCV regulation by employing inhibitors of epigenetic events.

**Results:**

The histone deacetylase inhibitors trichostatin A (TSA) and sodium butyrate powerfully stimulated JCV early and late transcription while the DNA methylation inhibitor 5-azacytidine had no effect. Analysis of JCV mutants showed that this effect was mediated by the KB element of the JCV control region, which binds transcription factors NF-κB p65, NFAT4 and C/EBPβ and mediates stimulation by TNF-α. Stimulation of transcription by p65 was additive with TSA as was cotransfection with transcriptional coactivators/acetyltransferase p300 whereas depletion of endogenous p65 by RNA interference inhibited the effect of TSA. EMSA with a KB oligonucleotide showed p65 expression, TNF-α stimulation or TSA treatment each caused a gel shift that was further shifted by antibody to p65.

**Conclusions:**

We conclude that JCV is regulated epigenetically by protein acetylation events and that these involve the NF-κB p65 binding site in the JCV control region.

## Background

JC virus (JCV) is a human neurotropic polyomavirus and is the causative agent of progressive multifocal leukoencephalopathy, PML, which is a fatal demyelinating disease of the brain that involves the cytolytic destruction of oligodendrocytes by JCV replication. PML lesions are multiple foci of myelin loss, which cause debilitating neurological symptoms and are areas of demyelination in the brain containing oligodendrocytes with viral nuclear inclusion bodies and bizarre astrocytes, which are also productively infected by JCV. The common underlying feature of PML is a severe weakening of the immune system, especially HIV-1/AIDS. Even after the introduction of combination anti-retroviral therapies (cART), PML still remains a problematic disorder associated with HIV-1/AIDS [1]. Despite the rarity of PML, the high prevalence (66-92%) of antibodies in human sera against JCV indicates that exposure to the virus is very common and begins in childhood and continues into middle age [reviewed in [2]. After the primary infection virus persists in a latent state and further sequelae only occur in people with severe immunosuppression where viral reactivation leads to PML. Many important aspects of the JCV life cycle and the pathogenesis of PML remain unclear including the nature of the latent state, the mechanisms whereby it is maintained and the regulation of restoration of viral transcription/replication when virus reactivates and causes PML.

JCV is a circular double-stranded DNA virus of the Polyomaviridae family [3] that was isolated in 1971 from the brain of a patient with PML [4]. It has two protein coding regions, which coordinate the viral life cycle: the early and late coding regions. These are transcribed in opposite directions starting from the Non-Coding Control Region (NCCR), which lies between them [5]. The NCCR functions as the promoter for both the early and late coding regions and also contains the viral origin of DNA replication. A variety of cellular transcription factors, some being glial cell-specific and others ubiquitous, bind and regulate the NCCR and these cellular factors, together with the viral early gene product large T-antigen (T-Ag) facilitate the JCV life cycle [reviewed in [6]. For example, we have described a site (the KB element) that is located on the early side of the origin of replication and binds the transcription factors NF-κB and C/EBPβ [7] as well as NFAT4 [8]. Since these transcription factors are regulated by signal transduction pathways that are controlled by extracellular cytokines, we have suggested that control of the latency/reactivation of JCV may be regulated by cytokines acting through the KB element. We have found that cytokines including TNF-α and IL-1β stimulate JCV early and late transcription and that this is mediated through the KB element [9].

In addition to the binding of transcription factors, the expression of genes can be regulated by post-translational covalent modifications of chromatin itself, which is known as epigenetic regulation. DNA within the cell nucleus, including the circular episomal viral DNA in JCV-infected cells, is packaged into a dynamic complex of DNA and histones as well as other non-histone proteins and RNA. Changes in chromatin structure can regulate the degree of compactness of chromatin and its availability to the transcriptional machinery, thus modulating transcription of chromatin in vivo [10,11]. A complex series of regulatory signals orchestrate the epigenetic status of chromatin including DNA methylation and histone acetylation. The association of DNA methylation with the silencing of gene expression is a well-established mechanism of eukaryotic transcriptional regulation [12]. Methylation of DNA is a post-replication process whereby cytosine residues in the dinucleotide sequence 5’-CG-3’ (CpG) are methylated. Experimentally, DNA methylation can be inhibited by 5-azacytidine (AZA), which can activate transcription of genes whose expression is suppressed by methylation. Typically, eukaryote genes have CpG islands rich in the CpGs located near the promoter [13]. However, the genome of JCV (strain Mad-1, [5]) is remarkably lacking in CpGs and contains only 6 CpGs in the 394 bp NCCR. In contrast to DNA methylation, histone acetylation is associated with transcriptional activation. Dynamic reversible acetylation of histones is a key part of the transcriptional process [10,14]. Acetylation of histones is catalyzed by histone acetyltransferase enzymes (HAT) and removal by histone deacetylases (HDAC). Both HAT and HDAC act not only on histones but also on nonhistone proteins including certain transcription factors. Experimentally, HDACs can be inhibited by trichostatin A (TSA) or sodium butyrate (SB), which can activate gene expression by increasing histone acetylation.

In this study, we investigate a role for epigenetic modifications in the regulation of Mad-1 JCV. We found that TSA and SB but not AZA robustly stimulated JCV transcription. This effect was mediated by the JCV NCCR KB element, was additive with cotransfected NF-κB p65 and was inhibited by p65 siRNA. Thus JCV is regulated epigenetically by acetylation events involving NF-κB p65 operating at the KB element of the control region.

## Results

### JCV early and late transcription are inhibited by the histone deacetylase inhibitors (HDACi) trichostatin A (TSA) and sodium butyrate (SB) but not by the DNA methylation inhibitor 5-azacytidine (AZA)

**Figure 1:**
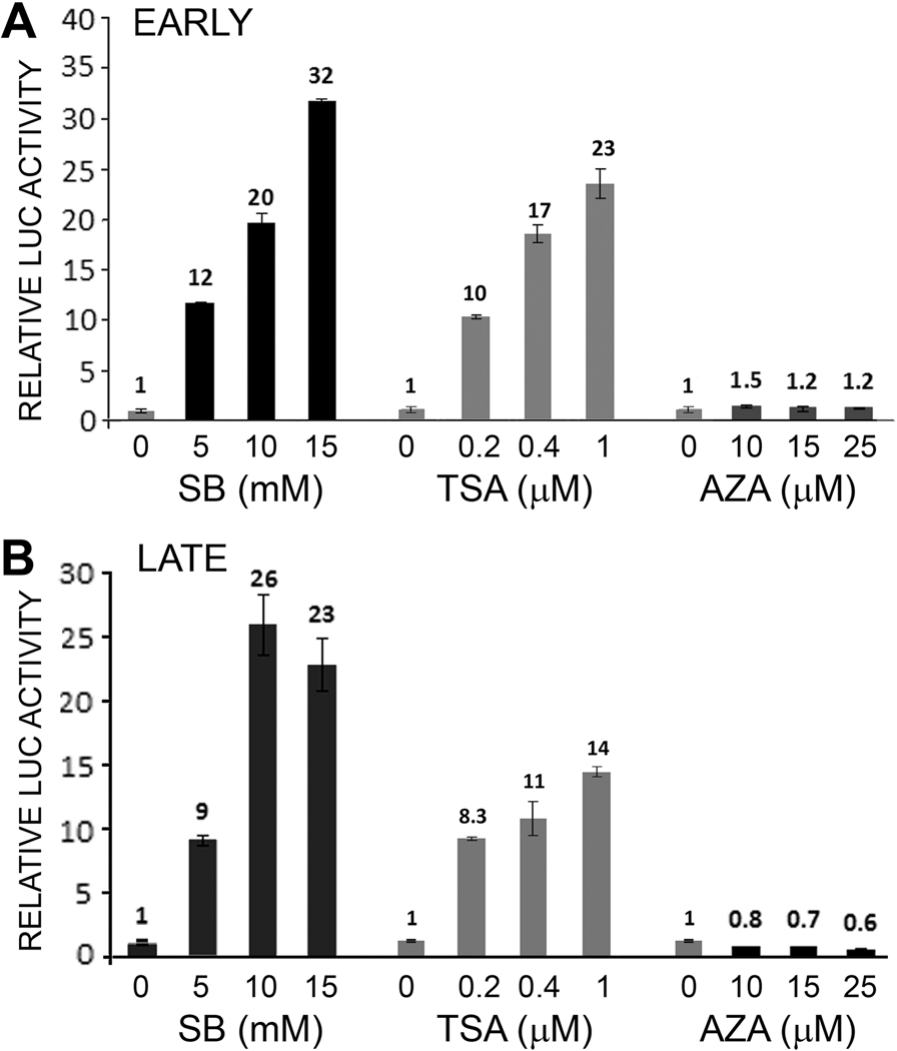
**Effect of the histone deacetylase inhibitors SB and TSA and the DNA methyltransferase inhibitor AZA on JCV early and late transcription.** TC620 human oligodendroglioma cells were transfected with the reporter plasmid JCV_E_-LUC **(A)** or JCV_L_-LUC **(B)** and treated with inhibitors, harvested and assayed for luciferase activity as described in Methods. Activities were normalized to untreated controls. The bar represents one standard deviation.

**Figure 2:**
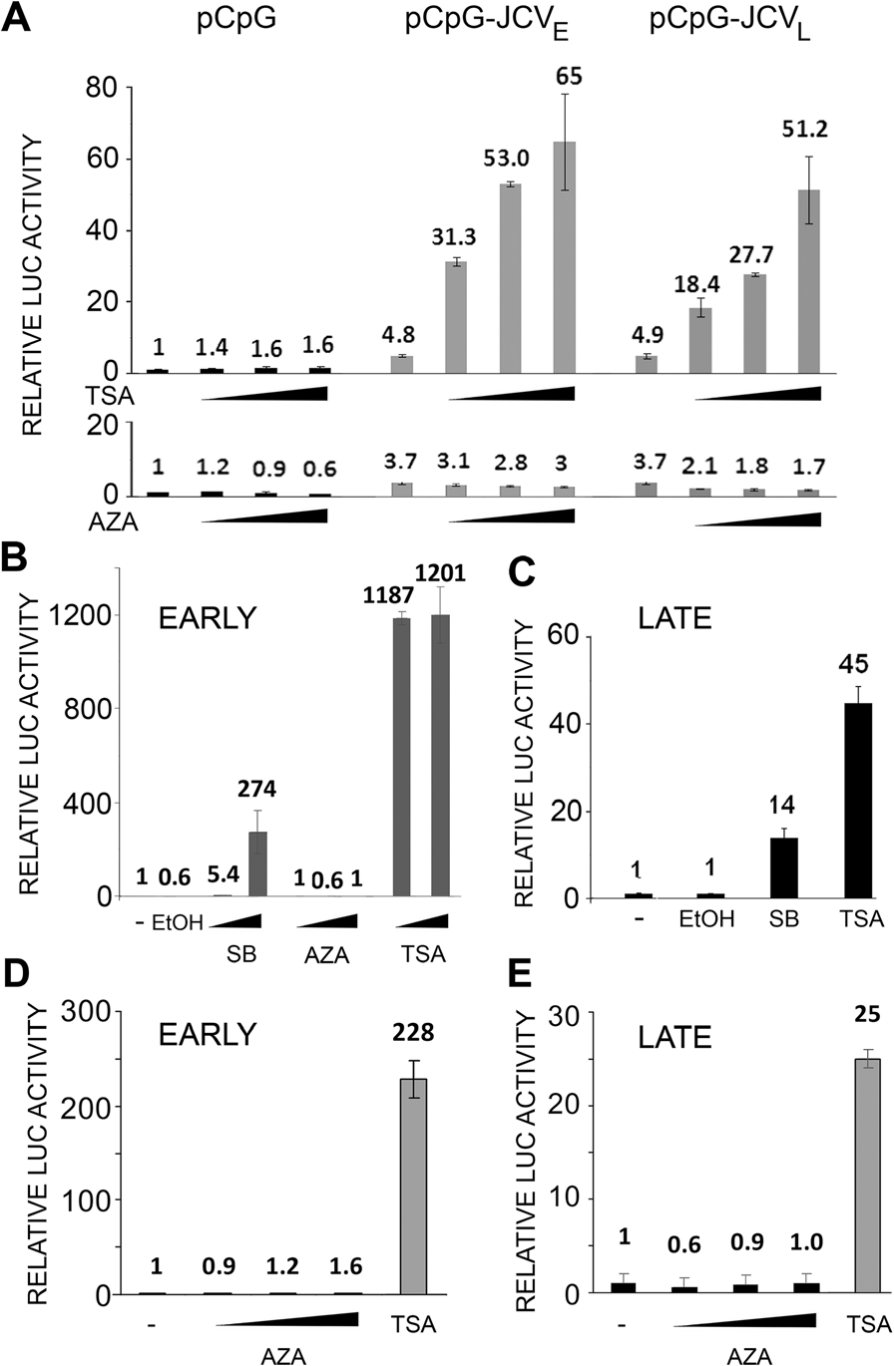
**Effect of the histone deacetylase inhibitors SB and TSA and the DNA methyltransferase inhibitor AZA on transcription of CpG-free reporter plasmids and on a TC620 clonal reporter cell line. A**. The JCV early and late promoters were cloned into the pCpG reporter plasmid and transfected into TC620 cells, which were then treated with inhibitors (SB – 5, 10 and 15 mM; TSA – 0.2, 0.4 and 1 μM AZA – 10, 15 and 25 μM), harvested and assayed for luciferase activity as described in Methods. Activities were normalized to untreated controls. The bar represents one standard deviation. **B**. A clonal cell line derived from TC620 by stable transfection with a plasmid encoding the luciferase reporter gene driven by the JCV early promoter [9] was treated with inhibitors (EtOH – ethanol vehicle control; SB – 5 and 10 μM; AZA – 10 and 15 μM; TSA – 0.5 and 1 μM), harvested and assayed for luciferase activity. Activities were normalized to untreated controls. The bar represents one standard deviation. **C**. As for B except using a stable clone with the late promoter (SB – 5 μM; TSA – 0.5 μM). **D**. As for B using a stable clone with the early promoter except treating with inhibitors for three days, adding fresh inhibitor everyday (Aza – 10, 15 and 20 μM; TSA – 0.5 μM). **E**. As for D except using a stable clone with the late promoter.

In our first experiments to investigate epigenetic regulation of JCV, TC620 human oligodendroglioma cells were transfected with luciferase reporter plasmids for the JCV Mad-1 early (Figure 1A) or late (Figure 1B) promoter and treated with different concentrations of SB, TSA or AZA. SB and TSA strongly stimulated (10-30-fold) transcription of both the early and late promoters while AZA was without effect. Since the reporter plasmids contain regions of high CpG content from the bacterial vector region and the luciferase gene, these could potentially become methylated and interfere with transcription. Thus, we also investigated transcription in a CpG-free reporter plasmid background using pCpGL-basic, which completely lacks CpG dinucleotides and was a kind gift from Michael Rehli, University Hospital Regensburg, Germany [15] into which we cloned the Mad-1 JCV NCCR in the early and late orientations to generate pCpGL-JCV_E_ and pCpGL-JCV_L_, respectively. These constructs gave essentially similar data (Figure 2A). In an earlier series of studies, we had generated a series of stable JCV early and late clonal reporter cell lines derived from TC620 [9]. We have investigated the effects of the epigenetic reagents on an early (Figure 2B) and a late (Figure 2C) reporter clone and found essentially the same results as for the transient transfection experiments. Even treating the early and late integrated reporter cells with Aza for 3 days, adding fresh inhibitor everyday, did not induce transcription (Figure 2D and 2E respectively).

### Mapping of the region within the JCV NCCR responsible for TSA inducibility implicates the KB element

**Figure 3:**
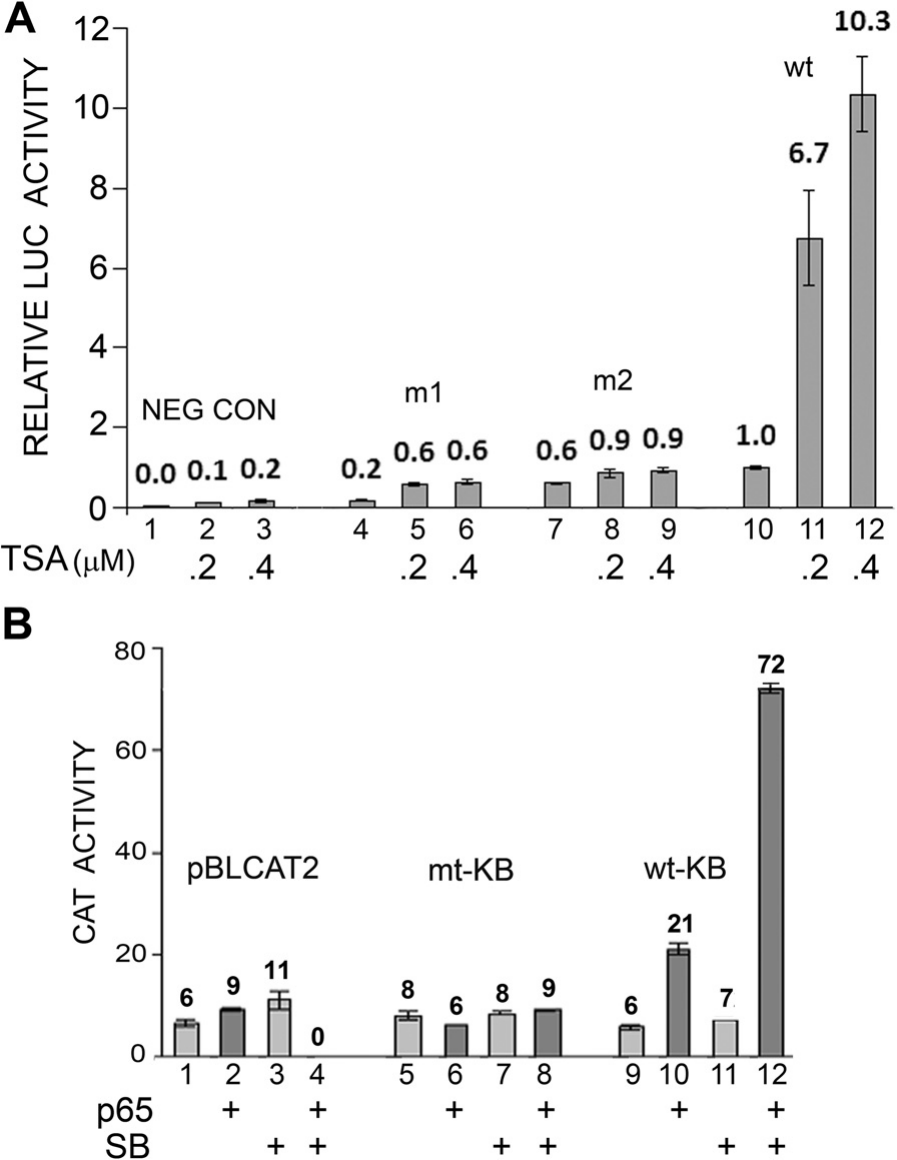
**Effect of TSA on JCV transcription by reporter constructs containing mutation in the KB element. A**. TC620 cells were transfected with JCV early reporter constructs containing mutation (m1 and m2) in the NCCR KB element and wild-type JCV early reporter, treated with TSA as indicated, harvested and assayed for luciferase activity. NEG CON – plasmid vector negative control. Activities were normalized to untreated wild-type control (lane 10). The bar represents one standard deviation. **B**. TC620 cells were transfected with pBLCAT2 or heterologous promoters containing either mutant or wild-type JCV KB elements, which we have previously described [9], treated with TSA as indicated, harvested and assayed for CAT activity. Activities are given in arbitrary units. The bar represents one standard deviation.

In our earlier studies, we had described a region within the JCV NCCR, called the KB element that is an important regulator of JCV early and late transcription, binds the transcription factors, NF-κB, C/EBPβ and NFAT4, and is responsible for the transcriptional response to cytokines such as TNF-α [7-9]. During these studies, we produced a variety of mutant and wild-type reporter systems to study JCV early and late transcription. To further analyze the effect of TSA, we examined two mutants, m1 and m2, which are base substitution mutants in the KB element of the JCV early promoter that abrogate the transcriptional response to NF-κB, NFAT4 and TNF-α [7-9]. As shown in Figure 3A, wild-type (wt) early promoter responded as expected but m1 and m2 failed to respond to TSA. Next, we examined heterologous promoters where the KB element (wild-type or mutant) was inserted upstream of the constitutive Herpes simplex virus thymidine kinase promoter in the CAT reporter plasmid pBLCAT2 [9]. As shown in Figure 3B, wild-type but not mutant KB conferred transcriptional inducibility on the promoter in the presence of p65 expression.

### NF-κB p65 and TSA cooperate to stimulate JCV early and late transcription

**Figure 4:**
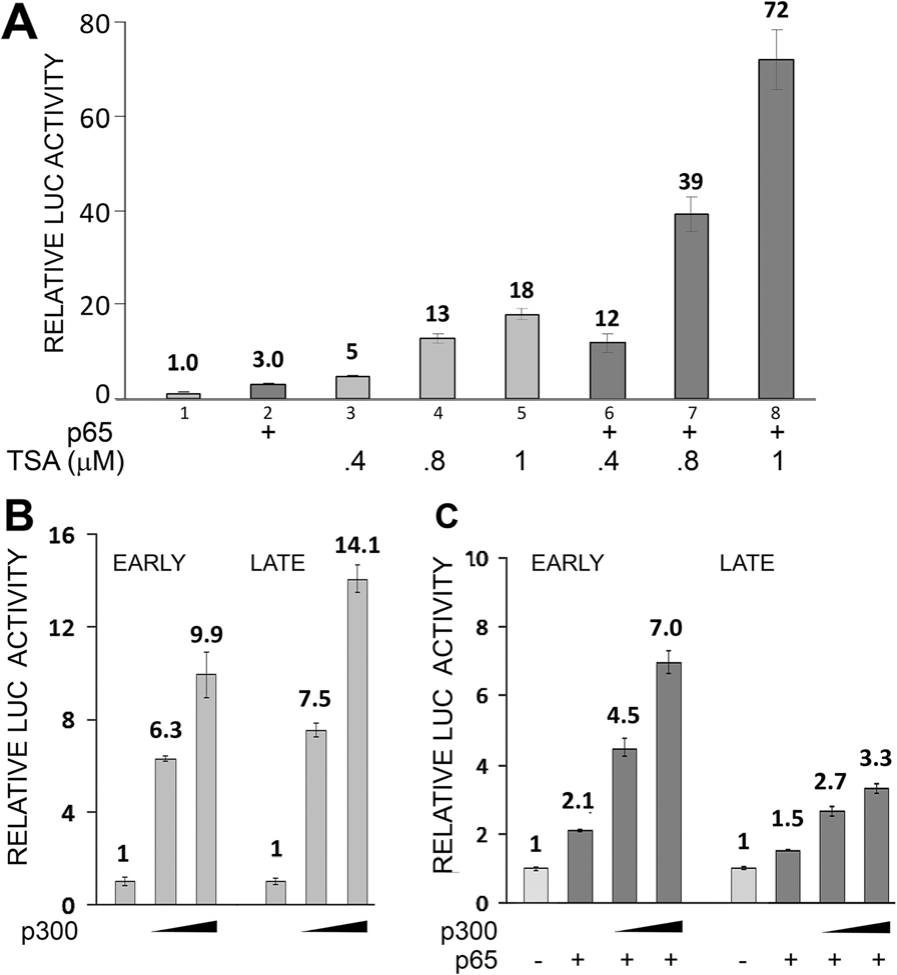
**Effect of TSA or coexpression of p300 on JCV transcription in TC620 cells expressing NF-κB p65. A**. TC620 cells were transfected with the reporter plasmid JCV_L_-LUC and NF-κB p65 expression plasmid and treated with TSA as indicted, harvested and assayed for luciferase activity. **B**. TC620 cells were transfected with the reporter plasmid JCV_E_-LUC (EARLY) or JCV_L_-LUC (LATE) and expression plasmid for p300 as indicated, harvested and assayed for luciferase activity. **C**. TC620 cells were transfected with the reporter plasmid JCV_E_-LUC or JCV_L_-LUC and expression plasmids for p65 and p300 as indicated, harvested and assayed for luciferase activity. Activities were normalized to untreated controls. The bar represents one standard deviation.

Since the activity of p65 has been reported to be increased by acetylation [16-19] and the mutational experiments indicated that the effect of TSA on early and late transcription may involve the KB element, we next examined the effect of expressing p65 on the TSA stimulation of the late promoter. The effect of TSA and transcription was markedly more pronounced in the presence of p65 (Figure 4A). For example, at 1 μM TSA transcription was four times higher in the presence of p65, compare lanes 5 and 8. Expression of p65 was shown by Western blot (data not shown). Similar results were obtained with early promoter (data not shown).

### Expression of p300 enhances JCV early and late transcription

Many transcription factors including p65 recruit the transcriptional coactivator p300, an acetyltransferase. p300 binds to p65 and catalyzes histone acetylation to open chromatin for the transcriptional machinery and acetylation of p65 itself to increase its activity [18,19]. We found that expression of the JCV early and late transcription (Figure 4B) and acted cooperatively with p65 (Figure 4C).

### Knockdown of p65 by RNA interference reduces the stimulation of JCV early and late transcription by TSA

**Figure 5:**
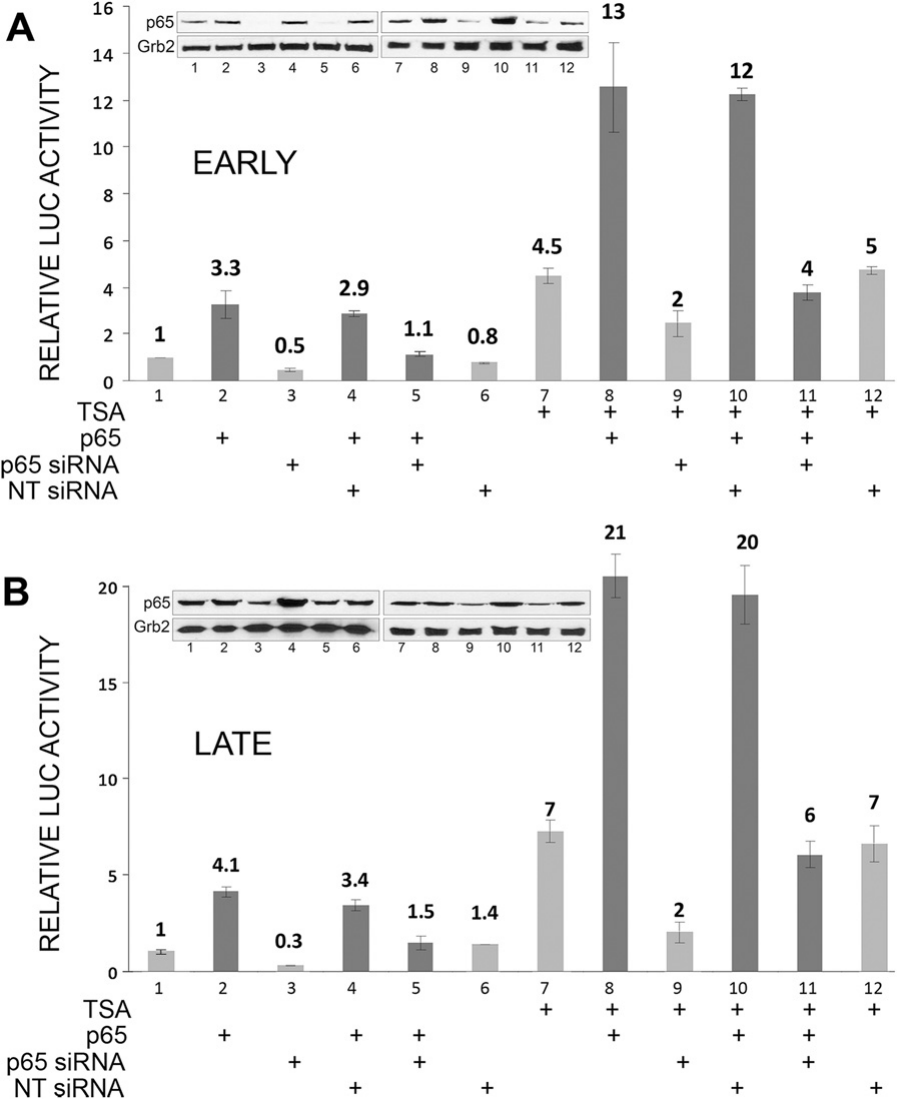
**Effect of RNA interference for NF-κB p65 on TSA-mediated stimulation of JCV transcription.** TC620 cells were transfected with the reporter plasmid JCV_E_-LUC **(A)** or JCV_L_-LUC **(B)** and expression plasmid for p65 and/or nontargeting (NT) or p65 siRNA and treated with TSA as indicated, harvested and assayed for luciferase activity. Activities were normalized to untreated controls. The bar represents one standard deviation. Western blots were performed for p65 and Grb2 (loading control) and are shown inset in each panel.

To further investigate a role for TSA in the mediation of the stimulatory effect of TSA on JCV transcription, we employed an RNA interference approached with an siRNA to p65 and a non-targeting (NT) control siRNA as was have described before [7]. As shown for early transcription (Figure 5A) and late transcription (Figure 5B), the stimulatory effect of TSA was partially reversed by p65 siRNA but not NT siRNA indicating the involvement of p65 in at least part of the effect of TSA on JCV transcription. Knockdown of p65 in the p65 siRNA-treated cells was verified by Western blot (shown inset in each panel).

### TSA induces an EMSA gel shift with a KB region oligonucleotide that co-migrates with that induced by p65 expression and TNF-α treatment and is supershifted by antibody to p65

**Figure 6:**
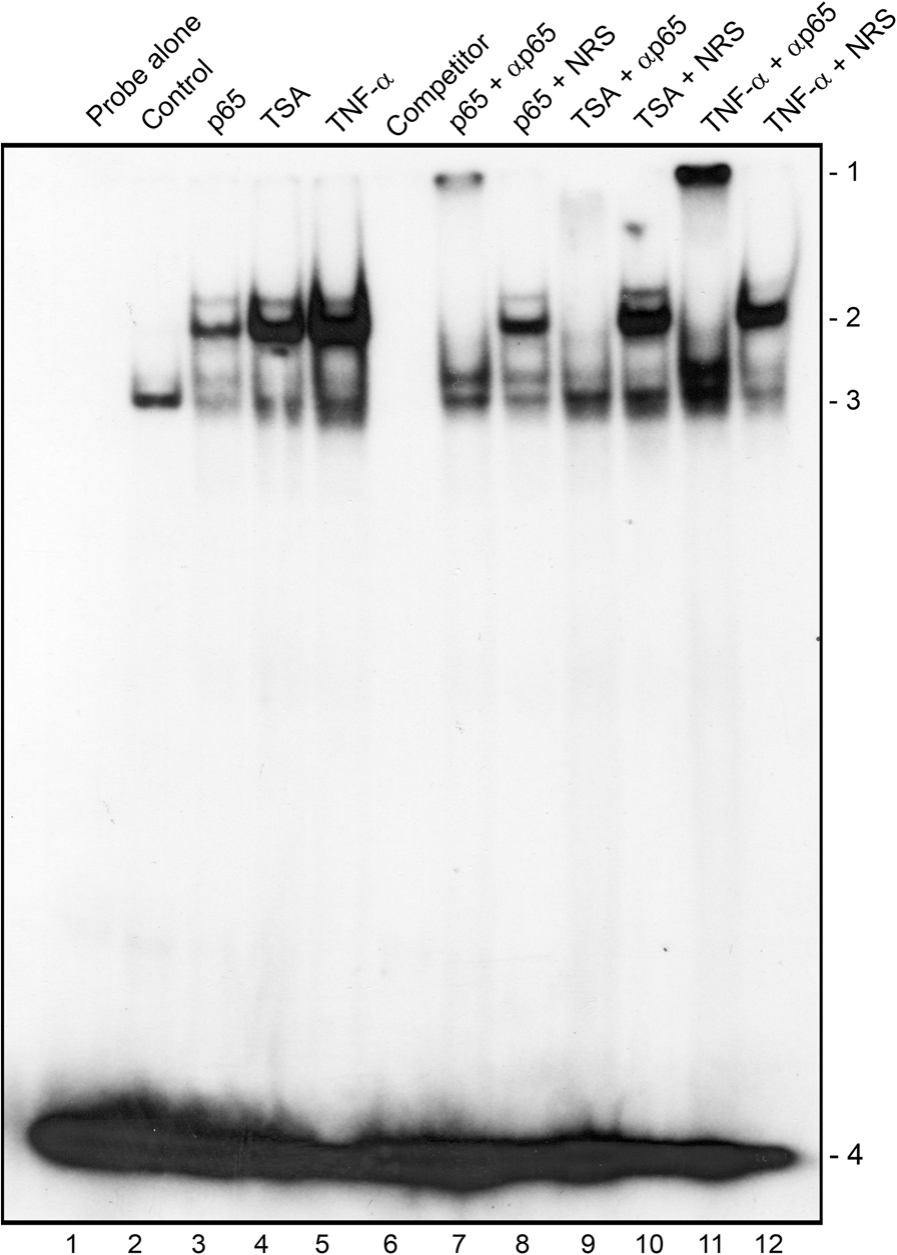
**Effect of p65 expression, TSA and TNF-α treatment on EMSA of the KB site.** An oligonucleotide corresponding to the JCV NCCR KB element was used to perform EMSA using nuclear extracts from untreated cells (Control) or cells transfected with expression vector for p65, treated with TSA overnight or with TNF-α for 30 min as described in Methods. For supershifts, antibody to p65 (αp65) or nonimmune rabbit serum (NRS) was added as indicated. Competitor – excess unlabelled probe was added. Unbound probe (loaded alone in lane 1) runs near the bottom of the gel (Position 4).

Next, we performed EMSA on nuclear extracts from cells that had been transfected with expression plasmid for p65, treated with TSA or stimulated with TNF-α for 30 minutes (Figure 6) and untreated control cell nuclear extracts. The probe used in this experiment was a double-stranded oligonucleotide corresponding to the JCV NCCR KB element that we have described previously [7,8]. As expected, ectopic p65 expression or short term treatment with TNF-α induced the appearance of a new band (lanes 3 and 4, position 2) and this new band contained p65 since antibody to p65 removed the band (lanes 7 and 11) whereas nonimmune rabbit serum did not (lanes 8 and 12) and induced the appearance of a lower mobility supershifted band (position 1). When cells were treated with TSA, this also induced a band in position 2 (lane 4) indicating that acetylation stimulation is associated with protein binding to the KB element and this band also contained p65 since antibody to p65 removed the band (lane 9) whereas nonimmune rabbit serum did not (lane 10) and induced the appearance of a lower mobility supershifted band albeit slightly below position 1 and more diffuse. Thus, inhibition of deacetylation is associated with increased binding of NF-κB p65 to its binding site in the JCV NCCR.

### TSA induces acetylation of histone H3 on lys-9 detected be ChIP assay

**Figure 7:**
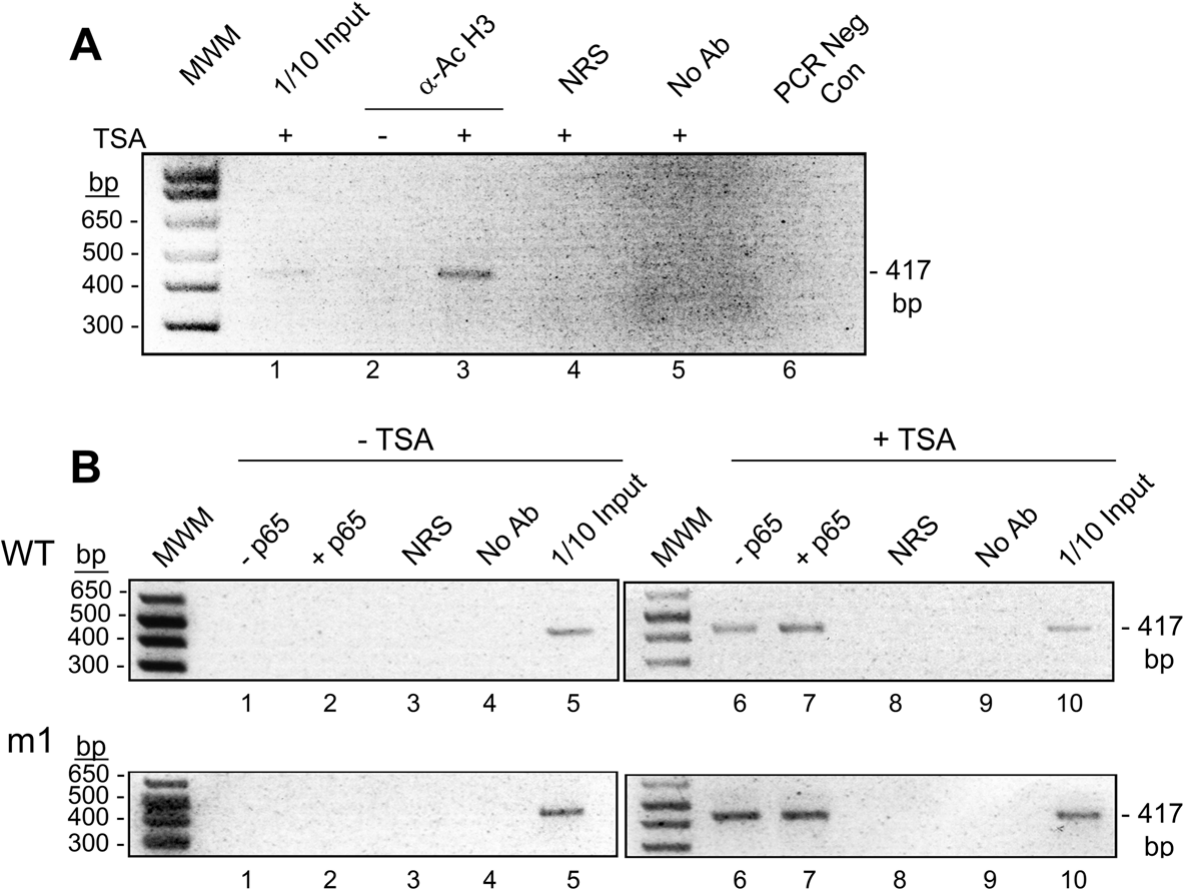
**Effect of TSA on histone H3 acetylation in the JCV NCCR. A**. TC620 cells were treated with or without TSA as indicated and ChIP assays performed as described in Methods. The positive control for the PCR was 1/10 of the input extract used in the IPs. MWM – molecular weight markers; α-Ac H3 – antibody to acetyl-histone H3 (Lys9); NRS – nonimmune rabbit serum; No Ab – no antibody added to the IP (beads alone); PCR Neg Con – PCR negative control (no template added). **B**. TC620 cells were transfected with p65 expression plasmid or control empty vector plasmid in combination with the JCV NCCR (wt or m1) and treated with or without TSA as indicated and ChIP assays performed as in Panel A.

To confirm changes in histone acetylation in viral chromatin induced by TSA, antibody to acetyl-histone H3 (Lys9) was used in IP for extracts from cells treated with and without TSA followed by PCR with primers that flank the NCCR (Figure 7A). A band was observed of the expected size for cells treated in the presence of TSA (lane 3) but not in its absence (lane 2). No band was observed with nonimmune rabbit serum (NRS, lane 2). These data confirm the expected changes in histone modifications induced by inhibition of histone deacetylase by TSA. In addition, we explored the possibility that p65 binding to the JCV NCCR might cause changes in histone acetylation. ChIP was performed on extracts from cells transfected with p65 expression plasmid or control empty vector plasmid in combination with the JCV NCCR (wt or m1) and treated with or without TSA followed by PCR with primers that flank the NCCR (Figure 7B). Again, a band was observed of the expected size for cells treated in the presence of TSA but not in its absence. The presence of transfected p65 did not have a significant effect. Thus, either binding of p65 to the wt JCV NCCR is without effect on histone acetylation or binding of p65 has only a local effect on histone acetylation that is below the level necessary for detection by this assay.

## Discussion

The state of latency/persistence is of central importance to the life cycle of JCV and when it is disrupted, the pathological events leading to PML ensue. While the site and molecular nature of viral latency/persistence are poorly understood, JCV is thought to persist in a number of organs including the kidney, bone marrow and brain [reviewed in [2,20]. In the kidney, JCV has an archetype NCCR configuration [21] and is likely undergoing active asymptomatic replication at a low level or episodically in the epithelial cells of the kidney tubules as shown by continuous shedding of the same strains of JCV [22]. On the other hand, JCV detected in the brain has a neurotropic configuration [23,24] and is likely to be within viral chromatin in a nonreplicating, nontranscribed state since JCV DNA can be detected but not expression of viral proteins [23]. Since, transcription of a given piece of DNA can be regulated by epigenetic modification to chromatin, we surmised that such regulation may occur for JCV.

Post-translational covalent modifications of chromatin, i.e., epigenetic changes, determine the openness of chromatin conformation and availability to the transcriptional apparatus, which can determine the level of gene expression of a region of DNA within the cell nucleus [10,11]. The major determinants of the epigenetic status of chromatin are DNA methylation and histone acetylation. Thus, in our initial experiments, we inhibited DNA methylation with AZA or enhanced acetylation of histones with HDACi, which would be expected to increase transcription if it was restrained DNA methylation or lack of histone acetylation respectively. We found that both JCV early and late transcription were greatly stimulated by the HDACi TSA and SB but not by AZA (Figure 1) indicating the importance of protein acetylation in JCV regulation but no involvement of DNA methylation. Since typically, promoter regulation involves large promoter-proximal CpG islands [13] and JCV contains only 6 CpGs in the NCCR [5], this is perhaps not surprising. Importantly, since our JCV early and late reporter constructs contained many extraneous CpGs from the luciferase and vector regions of the plasmids, these may potentially interfere with our analyses and so it was important to verify our data using a CpG-free reporter plasmid background. We used plasmids based on pCpGL-basic, which completely lacks CpG dinucleotides [15] and these constructs gave essentially similar data (Figure 2A). For some earlier studies, we had generated some stably transfected JCV early and late clonal reporter cell lines from TC620 cells [9]. When we investigated the effects of the epigenetic reagents, we also obtained the same results (Figure 2B & C) as for the transient transfection experiments.

Analysis of mutant JCV promoters implicated the KB element of the NCCR in mediating the effect of histone deacetylation inhibition since mutations in the element (m1 and m2) abrogated the effect (Figure 3A) and the effect of p65 on a heterologous promoter containing the KB element was potentiated by sodium butyrate (Figure 3B). Further, the stimulation of JCV late transcription by p65 was potentiated by TSA (Figure 4A) and siRNA to p65 inhibited the stimulatory effect of TSA on early (Figure 5A) and late (Figure 5B) transcription. It should be noted that there is one other report of JCV activation by HDACi I the literature [25] using the MH1 strain of JCV. In this report, deletion and site-directed mutational analyses of TSA-mediated activation indicated the importance of the enhancer region and an Sp1 binding site upstream of the TATA box, which is not present in the Mad-1 JCV NCCR [26]. Thus, it is possible that the mechanism of transcriptional induction by TSA may vary between strains of JCV.

From the gel shift data in Figure 6, it can be seen that p65 binding is induced by p65 overexpression (as expected), or TNF-α treatment, which activates the NF-κB signaling pathway (also expected) or by TSA. The TSA induction indicates that increased acetylation of p65, histones in the chromatin at the NF-κB site or both is sufficient to recruit NF-κB binding. By performing chromatin immune precipitation (ChIP) assays, we were able to show that TSA induced acetylation of histone H3 on lysine-9 within the chromatin of the JCV NCCR (Figure 7) but the assay was not sensitive enough to reveal if TSA changed the acetylation status of p65 using either acetyl-p65-specific antibody or anti-p65 immunoprecipitation followed by Western blot with anti-acetyl-lysine antibody (data not shown). Similarly, no effect of p65 on histone acetylation within the chromatin of the JCV NCCR was observed in the absence of TSA (Figure 7B) indicating that either it does not occur or, if it does, it is below the level necessary to be detected by the ChIP assay.

Taken together, our data suggest the region of chromatin at the NF-κB-binding site is involved in the stimulation of Mad-1 JCV transcription by HDACi such as TSA. The mechanism of this effect is still under investigation but it is possible that acetylation of histones and/or p65 via the p300 transcriptional coactivators/acetyltransferases is involved. NF-κB p65 is regulated by acetylation by p300 and CBP acetyltransferases, which principally target lysines 218, 221 and 310 [17-19]. Analysis of p65 mutants containing lys-to-arg substitutions indicates acetylation at K221 enhances DNA binding and impairs assembly with IκBα while acetylation at K310 is required for full p65 transcriptional activity [18]. In another study, data pointed to a role for K314/K315 in regulating p65 function [27]. As well as binding p65, the KB element binds C/EBPβ LIP [7] and NFAT4 [8], which act together with p65 to regulate JCV transcription. Hence, it is also possible that acetylation of these proteins is also involved in controlling transcription. C/EBPβ is functionally modified at several lysine residues but only K215/K216 is in the LIP domain [28]. There are no reports of NFAT4 acetylation but NFAT2 is acetylated [29]. In principal, acetylation of any of the three transcription factors, NF-κB p65, C/EBPβ and NFAT4, alone or in combination, may be responsible for activation of the JCV KB element by HDACi.

In conclusion, our data are consistent with a model where latent JCV is present in transcriptionally silent, deacetylated chromatin but can be activated by the action of transcription factors that act downstream from cytokines such as TNF-α and involve acetylation events. This is similar to latent HIV-1 provirus where marked transcriptional activation of the HIV-1 promoter also occurs in response to deacetylase inhibitors. Deacetylation events are an important mechanism of HIV-1 transcriptional repression during latency, whereas acetylation events are involved HIV-1 reactivation from latency [30]. Notably, HDACi (TSA and SB) synergized with both ectopically expressed p50/p65 and TNF-α treatment to activate the HIV-1 LTR [31]. While these findings with HIV could open new therapeutic strategies aimed at decreasing or eliminating the pool of latently HIV-infected reservoirs by forcing viral expression, our findings for JCV could open new therapeutic strategies for PML aimed at preventing viral expression and containing JCV in a latent state. Finally, at least ten human polyomaviruses are now known to exist [32] and it will be of interest to investigate if any of these are also regulated epigenetically.

## Methods

### Cell culture and plasmids

Culture of the human TC620 oligodendroglioma cell line was performed as we have previously described [9]. Stable clonal cell lines expressing luciferase under the control of the JCV early and late promoters were derived from TC620 as previously described [9]. Reporter constructs, JCV_E_-LUC and JCV_L_-LUC contained the JCV promoter from the Mad-1 strain linked to the luciferase gene in the early and late orientations respectively [9]. JCV_E_-LUC promoter mutants m1 and m2 contained mutations at two adjacent sites within the KB site of the early promoter and heterologous reporter plasmids, pBLCAT2-wt-kB and pBLCAT2-mt-kB contained wild-type and mutant KB elements respectively cloned into the CAT reporter plasmid pBLCAT2, which contains the constitutive Herpes simplex virus thymidine kinase (tk) promoter [9]. New reporters were generated based on a CpG-free luciferase vector, a kind gift from Michael Rehli, University Hospital Regensburg, Germany [15] by cloning the Mad-1 JCV NCCR was cloned into the BglII site of pCpGL-basic (pCpGL-JCV_E_ and pCpGL-JCV_L_). The expression plasmids pCMV-p65 and pCMV-LIP were described previously [7].

### Antibodies

The following antibodies were used for Western blot: Rabbit polyclonal anti-p65 (c-20, sc-372, Santa Cruz Biotechnology Inc., Santa Cruz, CA) and mouse monoclonal anti-Grb2 (610111; BD Biosciences, San Jose, CA). For EMSA, rabbit polyclonal anti-p65 (c-20, sc-372X, Santa Cruz) was used and for ChIP, rabbit monoclonal to acetyl-histone H3 (K9) (C5B11, Cell Signaling Technology, Danvers MA).

### Western blots

Western blot assays were performed as previously described [8]. Briefly, 50 μg of protein was resolved by SDS-PAGE, transferred to nitrocellulose, and immunoblotted with primary antibody (1/1000 dilution) and secondary antibody (1/10000 dilution). Bound antibody was detected with an ECL detection kit (Amersham, Arlington Heights, IL).

### Transient transfection and reporter assays

Co-transfection of reporter plasmids and expression plasmids were performed as we have previously described [7,9]. Briefly, TC620 cells were transfected with reporter constructs alone (0.5 μg) or in combination with the various expression plasmids for 48 h prior to. The total amount of transfected DNA was normalized with empty vector DNA. Treatment with epigenetic reagents was performed for 24 h prior to harvesting: SB – 0, 5 mM, 20 mM and 15 mM; TSA – 0, 0.2 μM, 0.4 μM and 1 μM; AZA – 0, 10 μM, 15 μM and 25 μM. Assays for luciferase and CAT were performed as previously described [7,9]. For RNA interference experiments, 200 nmol of Smartpool siRNA or control nontargeting siRNA (Dharmacon, Lafayette, CO) against p65 were transfected into cells 48 hours prior to transfection with the reporter plasmid as we have previously described [7].

### Gel shift assays

TC620 cells were transfected with p65 expression plasmid for 48 h, treated overnight with 250 nM TSA or treated 10 ng/ml TNF-α for 30 min and then harvested. Nuclear proteins were then extracted and 10 μg were incubated with 50,000 cpm of a γ-^32^P-labeled, double-stranded oligodeoxyribonucleotide probe as previously described [7]. The probe that was used in these gel shift experiments corresponded to the KB element: κB: 5′-aaaacaagggaatttccctggcctc-3′ (nts 5052–5078, Mad-1 JCV, GenBANK # NC_001699).

### Chromatin immune precipitation (ChIP) assay

TC620 cells were plated at a density of 1 × 10^6^ cells/ml in 100 mm dishes and the next day transfected with 3 μg of JCV_E_-LUC plasmid. The next day, cells were treated with 0.5 μM TSA for 24 hours and ChIP assays performed using the ChIP assay kit (Upstate Cell Signaling Solutions) as we have previously described [7]. Briefly, cells were cross-linked with formaldehyde and DNA sheared by sonication. After lysis, immunoprecipitation was performed with antibody to acetyl-histone H3 (Lys9) or nonimmune rabbit serum control as indicated. After DNA extraction, PCR was performed using the following primers which amplify a region spanning the entire JCV NCCR:

Forward: 5′-cctccctattcagcactttgtcc-3′ (Mad-1, 4989–5011).

Reverse: 5′-ggccagctggtgacaagcc-3′ (276–258).

PCR was 30 cycles (94°C for 30 s, 55°C for 30 s, 72°C for 30 s) then 72°C for 7 min.

## Abbreviations

AZA: 5-azacytidine; cART: Combination antiretroviral therapy; HAT: Histone acetyltransferase; HDAC: Histone deacetylase; HDACi: Histone deacetylase inhibitor; JCV: JC virus, polyomavirus JC; NCCR: Noncoding control region; PML: Progressive multifocal leukoencephalopathy; SDS-PAGE: Sodium dodecyl sulfate polyacrylamide gel electrophoresis; SB: Sodium butyrate; T-Ag: Larger T-antigen; TSA: Trichostatin A; wt: Wild-type.

## Competing interests

The authors declare that they have no competing interests.

## Authors’ contributions

HW and BW performed the experiments described in this study. KK and MKW conceived of this study. KK, MS, HW and MKW designed the experiments and interpreted the data. MKW processed and analyzed the data and wrote the manuscript. All authors read and approved the final manuscript.
